# Caregiver Mental Health Outcomes: Are There Differences Across Generations?

**DOI:** 10.1093/geroni/igaa057.082

**Published:** 2020-12-16

**Authors:** Susanna Mage, Laura Rath, Kathleen Wilber, Zachary Gassoumis

**Affiliations:** 1 University of Southern California, Leonard Davis School of Gerontology, Los Angeles, California, United States; 2 Archstone Foundation, Long Beach, California, United States; 3 University of Southern California, Chatsworth, California, United States

## Abstract

Informal caregivers are a critical component of support for the rapidly aging population. Previous studies have addressed the effects of caregiving on mental health. However, they have not focused on differences among generational cohorts of caregivers of older adults, i.e., Millennial (born 1981-1996), Generation X (born 1965-1980), Baby Boomer (born 1946-1964), and Silent Generation (born 1928-1945). As the Millennial caregiver population grows in parallel with older adults and their increased needs, we must better understand Millennial responses to caregiving. Millennial caregivers provide a similar intensity of care as Baby Boomers in terms of hours per week but are more likely to be fully employed (40+ hours per week or more). We used caregiver data from the nationally representative Centers for Disease Control’s Behavioral Risk Factor Surveillance System (BRFSS) survey from 2015-2017 to conduct negative binomial regression (n=50,745). Data analysis indicates that Millennial caregivers have an incidence rate ratio of 1.22 times more self-reported days of “stress, depression, and/or problems with emotions” compared to Generation X caregivers (p<0.01); 1.64 times compared to Baby Boomers (p<0.001); and 2.38 times compared to Silent Generation caregivers (p<0.001). Generational differences show that Millennial caregivers may have different needs than older generations of caregivers. Rather than assuming that the policies and interventions designed for older generations of caregivers will fit younger generations, implications of this work can help inform: 1) the design of programs to support caregivers’ mental health, and 2) policy considerations that address the unique needs of a younger caregiver population.

